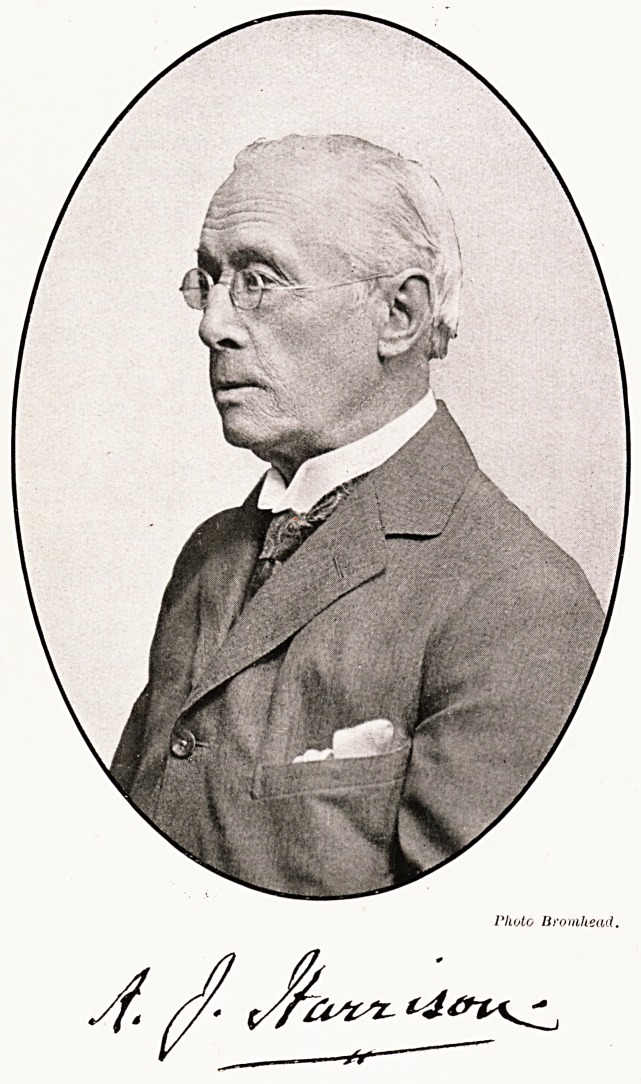# Alfred James Harrison

**Published:** 1924-04

**Authors:** 


*Photo Bromhead.*

A. J. Harrison

### ALFRED JAMES HARRISON, M.B. Lond.

It is granted to very few men to enjoy full activity and vigour for the long span of eighty-eight years, and to carry on their interests to the last with hardly any physical disabilities, yet this was the happy lot of our old colleague, Dr. Harrison. With the figure and gait of a man forty years younger, he carried on his usual pursuits and his daily walk of five miles almost up to the time of his death.

He was born in 1836, and was the son of Thomas Lichfield Harrison, of Belper. He became a medical student at Birmingham and in Guy's Hospital. Having taken the M.R.C.S. in 1858, the time of the Indian Mutiny, by the way, he was still too young to begin practice, and went for a voyage round the world on the clipper (sailing) ship *Percy*. In 1860, he was appointed Medical Officer to the Carey Street Dispensary, London, but went before long to the Rotunda in Dublin to study gynæcology, and took the London University M.B. in 1861. Then for a time he worked in Birmingham, where he was elected a Physician to the Children's Hospital and Lecturer on Materia Medica at the Sydney College, an institution soon after amalgamated with Queen's College.

In 1864 he took up private practice in Walsall, where he remained twelve years. He was on the staff of the Cottage Hospital there at the time when the celebrated Sister Dora had to do with the nursing arrangements. He appears to have taken an active part in public matters and was placed on the local bench of Magistrates. In 1865 he married Selina, the daughter of George Bradnock Stubbs, of Walsall, by whom he had ten children. At length, in 1877, he gave up his Staffordshire practice and retired to live at Failand Lodge, Clifton, but his usual love of work led him to seek election on the staff of the General Hospital, and he was appointed in November, 1879, to the somewhat arduous post of Assistant Physician. He then set himself to specialise in Dermatology, and by degrees he developed an important skin department at that Hospital, upon which he expended infinite care and skill, retaining the charge of it till 1904. Among other researches at this time he gave much attention to Tinea Tonsurans and Lupus, writing valuable papers on the former in 1885 and 1889 and on the latter in 1892. He devised a method by which a parasiticide drug could be carried to the deeper layers of the skin where the organism is buried, and in this he attained considerable success. His method has still many supporters. When Finsen brought out his Light treatment for skin diseases Dr. Harrison undertook a journey to Copenhagen and studied the new process in Finsen's clinic before carrying it out at home. In 1894 he was President of the Dermatological section of the British Medical Annual Meeting when Unna and other leading Dermatologists came to Bristol.

He had been appointed Lecturer on Toxicology in the Bristol University College in 1881, and carried this on for eighteen years. One of his chief recreations was found in the care of the Zoological Gardens, in which he took active part, and when elected President of the Medico-Chirurgical Society he charmed his hearers by an unexpected address on Comparative Pathology as illustrated by the clinical histories of his animal patients in the Gardens.

In 1899 he had the grief of losing his wife, and later on, as his family had become somewhat scattered, he undertook a voyage to New Zealand to visit his son who was in practice there. When the Great War broke out in 1914 he had given up most of his appointments, and was 78 years of age, but he determined to do his share. He took on once more the Skin Department at the Hospital and other forms of work to set free younger men, and finally went as a resident medical officer at a Mental Asylum in the district, a marvellous amount of toil for a man of his years.

In these notes we have only been able to refer to a few of his interests and labours, but his colleagues will always retain a warm memory of his genial manner and his devotion to his duties. He died on February 25th after a short illness. His remains were cremated at Woking after a service in St. Paul's Church, which he had attended for nearly fifty years.

BIBLIOGRAPHY.

"On Primary Endocarditis," *Brit. M. J.*, 1882, ii. 930–932.

"A New Method of Treating Tinea Tonsurans," *Brit. M. J.*, 1885, ii. 434.

"Further Researches on the Treatment of Tinea Tonsurans, illustrated by Micro-Photographs," *Brit. M. J.*, 1889, i. 465–467.

"Factitious Urticaria," *Bris. Med.-Chir. J.*, 1890, viii. 32–35.

"Cases of Rötheln and Red Rash which occurred at Clifton College in the summer of 1891," *Brit. J. Dermat.*, 1892, iv. 112–116.

"Local Treatment of Lupus with Sulphurous Acid," *Brit. M. J.*, 1892, ii. 298.

"On the Germ Theory in Dermatology," *Brit. M. J.*, 1894, ii. 262–264.

"Two Unusual Cases of Verruca Necrogenica," *Brit. J. Demat.*, 1895, vii. 362–368.

Presidential Address, "Comparative Pathology: a study in the gardens of the Clifton Zoological Society," *Bris. Med.-Chir. J.*, 1894, xii. 273–294.

(With W. K. Wills) "Remarks on the Light Treatment of Lupus Vulgaris and Erythematosus and Rodent Ulcer," *Bris. Med.-Chir J.*, 1903, xxi. 23–30.

(With W. K. Wills) "Light Treatment," *Bris. Med.-Chir* [illegible]., 1903, xxi. 303–309.

"Dermatitis from without, Dermatitis from within," *Bris. Med.-Chir J.*, 1906. xxiv. 325.

(With I. Walker Hall) "Fatal Enteritis in a Tiger caused by Physaloptera Præputialis," *Parasitology*, 1909, ii. 29–31.

## Figures and Tables

**Figure f1:**